# Microgreens Production: Exploiting Environmental and Cultural Factors for Enhanced Agronomical Benefits

**DOI:** 10.3390/plants13182631

**Published:** 2024-09-20

**Authors:** Shiva Dubey, Niamh Harbourne, Mary Harty, Daniel Hurley, Caroline Elliott-Kingston

**Affiliations:** School of Agriculture and Food Science, University College Dublin, Belfield, D04 V1W8 Dublin, Ireland; niamh.harbourne@ucd.ie (N.H.); mary.harty@ucd.ie (M.H.); daniel.hurley@ucd.ie (D.H.); caroline.elliottkingston@ucd.ie (C.E.-K.)

**Keywords:** microgreens, controlled environment agriculture (CEA), seed density, food security, nutritional profile

## Abstract

An exponential growth in global population is expected to reach nine billion by 2050, demanding a 70% increase in agriculture productivity, thus illustrating the impact of global crop production on the environment and the importance of achieving greater agricultural yields. Globally, the variety of high-quality microgreens is increasing through indoor farming at both small and large scales. The major concept of Controlled Environment Agriculture (CEA) seeks to provide an alternative to traditional agricultural cultivation. Microgreens have become popular in the twenty-first century as a food in the salad category that can fulfil some nutrient requirements. Microgreens are young seedlings that offer a wide spectrum of colours, flavours, and textures, and are characterised as a “functional food” due to their nutraceutical properties. Extensive research has shown that the nutrient profile of microgreens can be desirably tailored by preharvest cultivation and postharvest practices. This study provides new insight into two major categories, (i) environmental and (ii) cultural, responsible for microgreens’ growth and aims to explore the various agronomical factors involved in microgreens production. In addition, the review summarises recent studies that show these factors have a significant influence on microgreens development and nutritional composition.

## 1. Introduction

**Figure 1:**
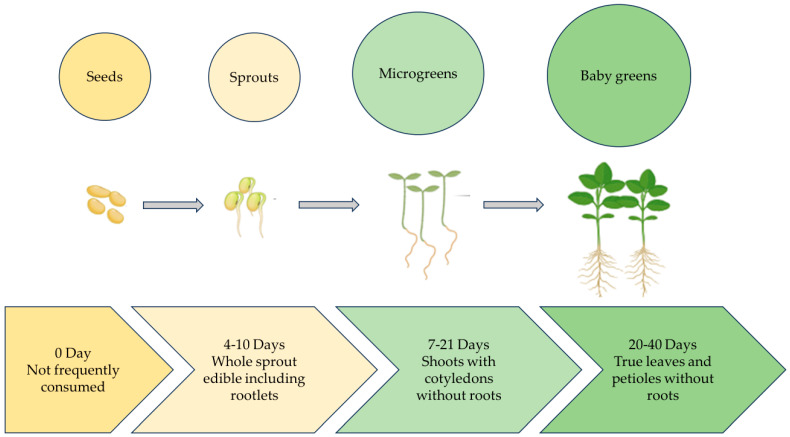
Early growth stages of plant.

One of the most challenging problems of the 21st century is the issue of food insecurity linked to global warming. One of the main objectives of urban farming globally is to feed the world’s steadily growing population [1]. According to the Urban Agriculture Committee of the Community of Food Safety Coalition (CFSC) (2003), urban farming produces and disperses food items in metropolitan cities using plant and animal husbandry [2]. Microgreens are defined as small plants with two fully developed cotyledon leaves, a pair of small true leaves, and a central stem [3]. Microgreens are an emerging crop and are tender seedlings harvested at the emergence of the first true leaves [4]. Microgreens were initially used in the late 1980s by high-end restaurant chefs in San Francisco, USA [5]. In contrast, sprouts are germinated seeds that represent the most diminutive and youngest of salad crops. The main distinction between sprouts, microgreens, and baby greens (later stage of microgreens) is the size of the plant and growing time [4]. Microgreens are harvested later than sprouts but earlier than baby greens [6]. Microgreens are harvested as soon as their newest leaves emerge, while baby greens are often harvested at 5 to 10 cm in height or 15 to 40 days from seed germination (Figure 1). Microgreens and sprouts also differ in their chemical composition [7]. More than twenty studies have defined them with respect to their growth and harvest period [6]. Microgreens are delicious, fresh, flavourful, and packed with nutrients; these tiny vegetables grow quickly, making them perfect for growing indoors [8]. Several research papers have acknowledged the importance of the health benefits of microgreens. Their rich nutrient profile includes high concentrations of antioxidants, minerals, and vitamins; it has been shown that bioactive compounds are present at higher levels of bioaccessibility in *Brassicaceae* microgreens [9,10], which can contain concentrations of β-carotene and vitamins (C, E, K) up to forty times greater than their mature counterparts [9]. Nutrient-rich microgreens can be grown in sustainable settings by using natural light and recyclable growth media [6]. In the last decade, a shift has occurred in food preferences. Consumers are inclined towards healthier and more nutrient-rich food. Recent statistical data and market research demonstrated a continuous increase in microgreens demand globally due to their nutritional properties and quick adoption in indoor and vertical farming [11]. Microgreens are suitable for urban farming and can also be grown at home, or they can be produced in large quantities in a CEA settings. Because they may be cultivated indoors and are available all year round, which contributes to their agronomical benefits, farmers can grow microgreens in spaces ranging from small greenhouses to large farms [12]. In order to cultivate plants for food production during NASA space missions, CEA technologies were first introduced in the 1970s [13]. Subsequently, microgreens have been investigated as an additional food source for astronauts on board the space station [12]. Microgreens offer a viable way to supplement astronaut meals with nutrient-dense fresh food in space settings. Their capacity to adapt to regulated settings, short development cycles, and high nutritional value make them perfect space candidates [14]. A study presents the development and testing of customised hardware for harvesting microgreens in microgravity, optimizing the process for an efficient and contamination-free system [15]. Although microgreens have significant advantages for space missions, there are still issues with streamlining their production and harvesting procedures to guarantee efficiency and sustainability in space habitats. Previous studies have primarily focused on the nutritional profile of microgreens [16,17,18]. However, optimisation of various factors such as light, humidity, temperature etc. requires further scientific attention to maximise microgreens quality, nutrient profile, and yield. This study provides new insight into two major categories, (i) environmental and (ii) cultural factors, responsible for microgreens’ growth and aims to contribute to research by exploring and elucidating the various agronomical factors involved in microgreens production. This study provides useful information for customising microgreens production to the growing demand for nutrient-rich, high-quality food on a worldwide scale. This will help to ensure food security and sustainable agricultural output.

## 2. Optimising Growing Conditions of Microgreens

**Figure 2:**
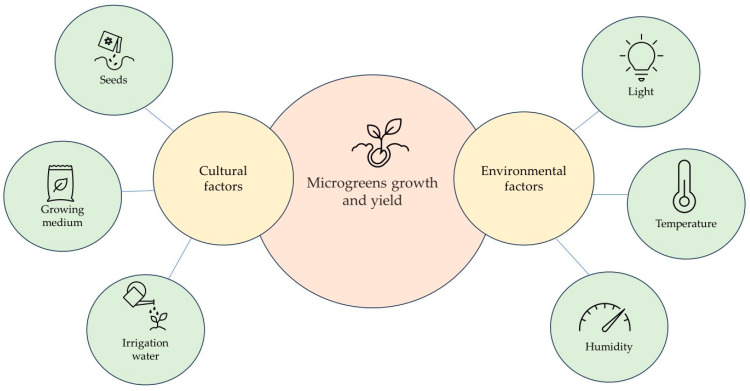
Cultural and environmental factors affecting microgreens’ growth and yield.

The growth of microgreens can be enhanced through the optimisation of various factors, including humidity, light (photoperiod, quantity, and quality), seed density, substrate, and temperature (Figure 2). These different growing conditions can influence nutrient content quality and yield. Previous studies have explored the effect of light on phytochemical synthesis and yield [3,19,20,21,22].

### 2.1. Seed Density and Quality

The literature on microgreens has highlighted the importance of seed density due to its direct influence on microgreens’ growth. Choe, Yu and Wang [7] explained a linear relationship between seed density and fresh weight yield. However, a decrease in individual shoot fresh weight shows an inter-species competition for limited resources. Similarly, Lee et al. [23] reported the same relationship between beet and chard microgreens seed density and yield. Later, Murphy and Pill [24] observed similar results in arugula (rocket). Seed density is a crucial step from an economical and commercial point of view to obtain optimal growth results. A recent study recorded a positive correlation between seed density and yield of mung bean and lentil microgreens. However, poor quality microgreens were observed once the seed density surpassed the optimal threshold [25]. The optimum seeding density is highly species specific and is typically determined by the mean of seed weight and germination percentage (%) [24]. A strong relationship between seed density and yield has been reported in Indian lentils and mung bean microgreens. The optimum seed density for lentils and mung beans was three seeds/cm^2^; however, for Indian mustard, eight seeds/cm^2^ is optimum. Similarly, another study by Paradiso et al. [26] analysed microgreens from three species (*Brassica oleracea* L. Group *Cichorium intybus* L., *italica* Plenk, and *Lactuca sativa* L. Group *crispa*) of locally available seeds (Italy). The seed density used for *B. oleracea* genotypes was four seeds/cm^2^ while for *C. intybus* and *L. sativa* it was three seeds/cm^2^. Therefore, seed density directly depends upon the seed size of a microgreens species [25]. In addition, few studies have demonstrated the effectiveness of seed treatment on microgreens quality [23,27]; for example, soaking seeds in zinc solutions (e.g., ZnSO_4_) increases the amount of zinc in microgreens, enhancing their nutritional profile while preserving other micronutrient levels [28].

### 2.2. Substrate

Growing media has a significant effect on the growth of microgreens. Di Gioia et al. [29] concluded that growing media is critical to microgreens’ yield, quality, and microbiological quality. Microgreens can be grown on soil or on alternative growing media like clay, coco coir, copolymer fabric, fleece, glass wool, grape pomace, gravel, hemp, jute, paper straw, perlite, rice husks, rockwool, sand, sawdust, sugar cane fiber, tree bark, vermiculite, and zeolite [29,30,31]. Other studies have determined the impact of substrate on nutritional quality and yield of microgreens (Table 1). The hydroponic system uses mineral fertiliser solutions and abundant oxygen sources to grow plants crops without the need for soil. The most frequently hydroponically grown crops include tomatoes, lettuce, spinach, strawberries, cucumbers, melons, eggplant, peppers, and herbs (including basil, cilantro, and rosemary) [32]. Some studies demonstrate a higher concentration of phytochemicals and a lower concentration of potassium (K) in hydroponically grown microgreens, making them suitable for consumption by renal dysfunction patients [33,34]. The selection of a suitable growing media for microgreens can depend on the physical and chemical properties listed below [29,35].

**Figure 3:**
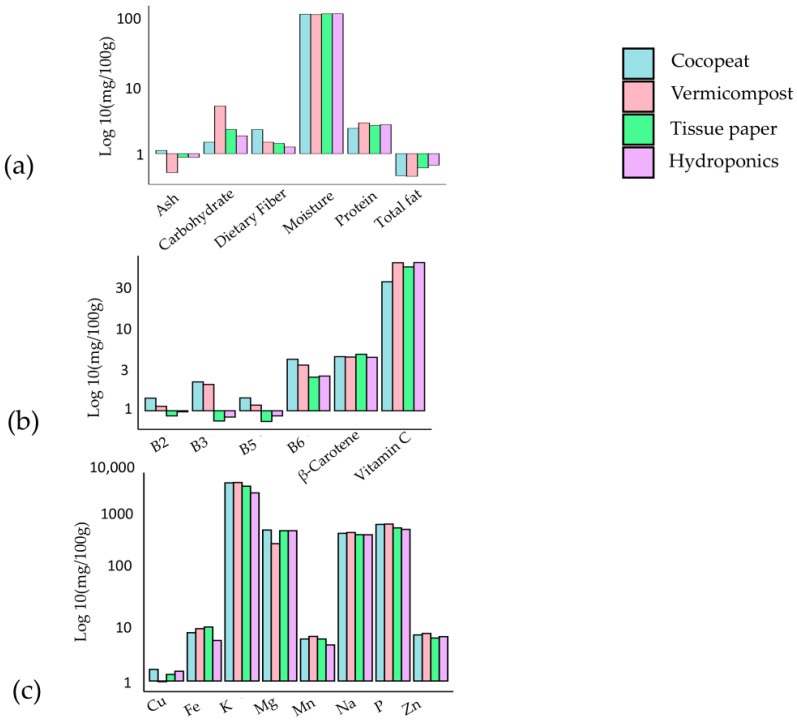
Nutrient composition of Pak choi on four different types of media: (**a**) macro-nutrients, (**b**) micro-nutrients, and (**c**) minerals [48].

The investigation assessed the nutrient composition of Pak Choi cultivated in four distinct media types for macro, micro-nutrients, and minerals (see Figure 3a–c, respectively). The hydroponic medium exhibited a high moisture content (92.85%) and total fat content (0.69%), indicating effective water retention and fat synthesis. Significant improvement in protein content (2.27%) was observed in the coco peat medium, along with substantial levels of vitamins B5 (1.46 mg) and B6 (4.56 mg). The vermicompost medium displayed increased carbohydrate content (4.68%), elevated vitamin C level (78.24 mg), and noteworthy concentrations of sodium (871.69 mg) and potassium (8561.74 mg). Moreover, the coco peat medium provided notable levels of potassium (1262.37 mg) and copper (1.70 mg). These findings emphasise the impact of growth media on the nutritional composition of Pak Choi, allowing for customised cultivation practices to fulfill specific nutritional and market demands [48]. Further investigation is imperative to comprehend the mechanisms underlying nutrient accumulation in various media, facilitating the optimization of Pak Choi cultivation. Combining substrates that use organic materials like coco coir mixed with soil can accelerate the risk of soil-borne diseases, which may jeopardise the health of microgreens [49]. These studies indicates that selection of a substrate has a major influence on sustainability, yield, and nutritional quality of microgreens. Natural fibre substrates such as jute and coconut fibre provide sustainable options with comparable yields and quality, although peat is still a popular choice [49]. Additionally, synthetic and compost-based substrates also represent viable options, each with unique benefits and drawbacks.

#### 2.2.1. Physicochemical Properties of Growing Media

##### Physical Properties

The primary physical characteristics of growing media, which include dry bulk density (BD), total pore space (TPS), particle density (PD), air capacity (AC), and water-holding capacity (WC), are crucial for determining their suitability for plant growth [29,50].

##### Chemical Properties

The main chemical properties of growing media are pH, electrical conductivity (EC), and organic matter (expressed as a percentage on a dry weight basis), which are critical factors for plant growth [51].

### 2.3. Water

The source and the method of irrigation water play a crucial role in fresh produce food safety. Microgreens can be cultivated using various irrigation methods, each with advantages and challenges. Subsurface irrigation is favored for its lower contamination risk, while overhead irrigation is still widely used despite its drawbacks [52]. The integration of technologies such as moisture sensors can further enhance irrigation practices, making microgreens production more efficient and sustainable [53,54]. A similar study reported the significant impact of irrigation practices on microgreens production, with sensor-based systems improving water use efficiency by up to 30% compared to gravimetric methods [55]. Outdoor transportation exposes water to soil bacteria and parasites while pipes expose the water supply to biofilms. The presence of *Escherichia coli* O157:H7 has been shown to multiply in both soil-substitute and hydroponics, whereas higher levels were observed in hydroponically grown microgreens [56]. Drip irrigation reduces the risk of contamination compared to overhead spray irrigation due to limiting exposure of the edible portion of the plants to water [57,58]. Irrigation methods can influence crop vulnerability to microbial pathogens mainly by influencing the amount of water content in plant tissues. Therefore, over-irrigation can lead to a high level of moisture in leaves, making plants prone to phytopathogens and exacerbating the severity of diseases [59].

### 2.4. Light

Plants may adapt to changes in light conditions, either by using radiation as a source of energy for photosynthetic processes or by interpreting it as a signal that is used by a network of photoreceptors with precise wavelengths to control photo morphogenetic reactions [60,61,62]. LED lights are the most effective and versatile artificial lighting systems for advanced CEA facilities. They offer several benefits over previous lighting options, including low energy consumption, minimal heat output, rapid response time, and the ability to be customised, as well as a long lifespan and a broad range of narrowband-emitting diodes [63]. The optimal range of light conditions (quality and quantity) induces photosynthetic activity and phytochemical production [10,64].

#### 2.4.1. Light Quantity

**Figure 4:**
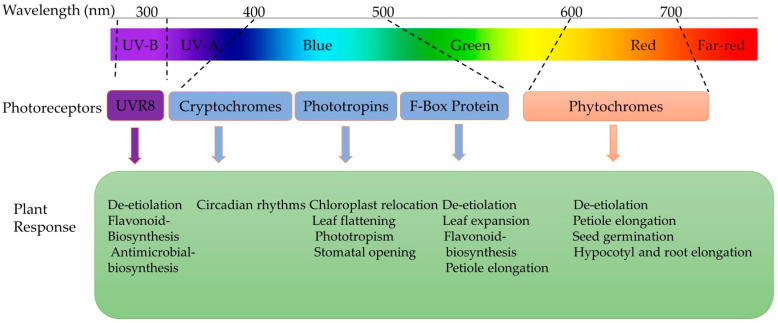
Different plant receptors responsible for plant responses under different spectrums of light [60,63,69].

Light signals are first perceived at the leaf level and then transduced to particular target systems, triggering molecular reactions that regulate metabolic processes (Figure 4) [63]. In the literature, the most frequently used photosynthetic photon flux density (PPFD) of light intensities ranges from 100 to 300 µmol·m^−2^·s^−1^ to produce microgreens [65]. A balanced light spectrum with a combination of blue, green, and red light at an intensity of 150 μmol m^−2^ s^−1^ PPFD is recommended for optimal microgreens’ growth in space as it promotes a compact architecture, enhances the nutritional value, and minimises energy requirements in comparison to higher light intensities [14,66]. On the other hand, in a controlled environment, overexposure to LED lights can have adverse effects on vegetables due to photodamage [67], and a higher light intensity can result in photoinhibition [68]. However, there is a lack of extensive research on the minimum PPFD required for microgreens production.

#### 2.4.2. Light Quality

Light quality (wavelength composition) influences plant responses and causes physiological and developmental changes [70]. Photoreceptors detect specific wavelengths such as blue (B, 445–500 nm), green (G, 500–580 nm), red (R, 620–700 nm), and far red (FR, 700–775 nm) (Figure 4). Meanwhile, specific photoreceptors are responsible for perceiving ultraviolet (UV) radiation, including UV-A (315–380 nm) and UV-B (280–315 nm) types [71]. However, the desirable nutrient profiles of microgreens can be manipulated by red, blue, or a mixture of both lights as they enhance photosynthesis and regulate plant metabolism more than white light [72,73,74].

### 2.5. Temperature

Temperature can significantly affect microgreens’ quality, shelf life, and sensory quality. The optimal temperature reported in previous studies can vary from 17 to 20 °C night/day [73,75]. Optimal atmospheric and soil temperatures are required for the growth and development of microgreens. Temperature has an influence on the different developmental stages of plants. The vegetative stage demands a greater optimal temperature than the reproductive stage [76]. Microgreens production efficiency is influenced by the production temperature. A linear rise in temperature from 14 °C to 22 °C can significantly decrease the day of harvest by 35–40% [6]. Once microgreens are harvested, an excessively high temperature can disturb morphology, physiology, and metabolic rate, which can lead to poor nutritional quality. A study by Xiao et al. [77] suggests that storage temperature can also impact morphology, microbial growth, and membrane integrity, and they reported that a storage temperature of 1 °C can maintain shelf life and quality. Microgreens growth is greatly impacted by the vapor pressure deficit (VPD) under different temperatures. Studies show that optimal VPD values can improve microgreens’ biomass yield and nutritional quality; these results are further influenced by particular temperature conditions. Spinach microgreens in controlled settings showed a 71% increase in dry weight, demonstrating the significance of VPD regulation in optimizing growth potential [78]. Another example of temperature influence has been reported in arugula microgreens; these microgreens exhibited greater elongation at 18 °C than at 28 °C, suggesting a complex relationship between light, temperature, and VPD [79]. Similarly, mustard microgreens show the least amount of deterioration over 14 days at 5 °C storage temperature [80]. This also suggests that storage conditions are as important for maintaining quality as VPD during growth.

### 2.6. Relative Humidity (RH)

RH influences water retention and photosynthesis, making it a crucial parameter for microgreens growth. Excessive humidity can affect both quality and safety of produce. Data trends suggest that the most used relative humidity is 50–70% [73,81]. An early study by Ford and Thorne [82] demonstrated the atmospheric humidity effect on kale, sugar beet, and wheat, showing that as humidity increased, the number of cells increased, resulting in a significantly larger leaf area in both kale and sugar beet. The correlation between relative humidity and VPD is another critical factor to consider in optimising the growth of microgreens as VPD is inverse to RH. Higher VPD accelerates the rate of transpiration in plants [78]. A study reported that higher VPD (1.76 kPa) improved phytochemical levels in lettuce; however, lower VPD (0.69 kPa) showed increased biomass, suggesting that VPD manipulation can improve yield and nutritional quality [83]. Although controlling VPD can positively contribute to the growth of microgreens, it is important to understand that high humidity can risk their quality [84,85]. Therefore, a balanced approach is required to maximise plant health and quality.

### 2.7. Genetic Traits Influencing Growth and Yield

Several varieties of microgreens are offered at the commercial scale; however, most of the research comprises a limited number of varieties, and the most studied taxa belong to the *Brassicaceae* family, with a lesser emphasis on the *Chenopodiaceae* family [19]. Genetic traits can directly impact the chemical composition and quality within the same taxa. Xiao, Lester, Luo, and Wang [9] listed differences in the phytonutrient profile of 25 genotypes belonging to 19 different taxa of commercially available microgreens. Jones-Baumgardt et al. [86] have reported unique growth and yield responses in four microgreens genotypes, arugula, kale, mustard, and sunflower, under different supplemental light levels. Although an environmental impact on the traits of microgreens is evident, genetic variability in this context has received limited scientific attention to date. Di Bella et al. [87] confirmed that growth-related traits of microgreens are significantly influenced by genotype.

### 2.8. Fertiliser

Instead of heavily relying on fertilization, many farmers employ commercially supplied peat lite soils with a higher nutritional richness. Microgreens require very little fertiliser because of their short growth period. However, the use of fertilisers can boost growth and nutritional content [88]. According to research finding, conventional or unconventional fertilisers, including spent brewer’s yeast, can be applied to enhance plant growth [89]. Specifically, rye showed improved biomass and hypocotyl length when treated with 50% volume concentration of discarded brewer’s yeast and 100 mg L^−1^ of ascorbic acid [89]. A study investigated the macro- and micro-nutrients in seventeen microgreens species selected from seven families. This study reported that among the primary macro-nutrients in microgreens, nitrogen (N) and potassium (K) make up the largest proportions, accounting for 38.4% and 33.8% of total macro-nutrients, respectively. In micro-nutrients, Fe was the most prevalent, with Zn, Mn, B, and Cu following in order of abundance. Sunflower and scallion were identified as rich sources of Cu and Zn. However, other species might benefit from supplementary fertilization to improve their micro-nutrients levels [90]. A commonly used method that involves applying calcium nitrate fertiliser before planting and giving a liquid fertiliser treatment after planting has increased fresh weight by about 20% despite the short 15-day growing period. Research on arugula microgreens supports this approach, suggesting that applying a pre-plant fertiliser and providing nitrogen fertilisation at 75–150 mg L^−1^ at different stages of growth can be beneficial [24,91]. Chinese kale microgreens can also be successfully cultivated using Hoagland’s solution at half strength. However, in this experiment, plant growth was allowed to continue beyond the typical 30-day period for microgreens [92]. On the other hand, researchers observed that the Chinese kale responded differently to light regimes than the 21-day-old brassica microgreens. Mizuna, arugula, and mustard microgreens did not have significantly higher fresh weights at higher fertiliser concentrations [93]. Supplementing with a general-purpose soluble fertiliser can raise fresh shoot weight and nutrient concentrations in a variety of microgreens species, including broccoli, cabbage, radish, kale, and pea, but it can also lower levels of calcium, magnesium, and manganese [94,95]. Fertilisers are dose-dependent in their ability to help microgreens growth; therefore it is important to avoid applying too much and squandering it during germination.

## 3. Nutritional Profile and Sensory Attributes

**Figure 5:**
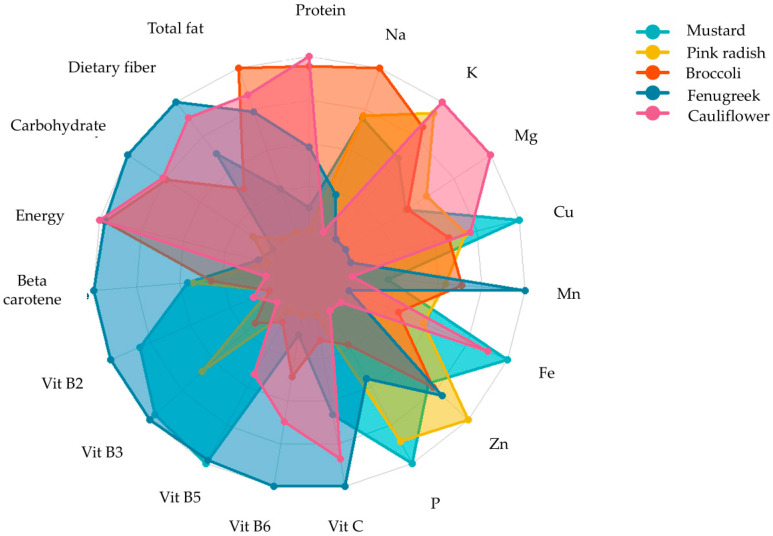
Nutritional profile of five microgreens grown on vermicompost [11].

Research indicates that microgreens contain a higher quantity of phytonutrients such as ascorbic acid, β-carotene, α-tocopherol, and phylloquinone than mature plants. They are also rich in minerals, such as potassium, calcium, magnesium, iron, and zinc, and essential elements, such as vitamin C, phenols, and glucosinolates [96]. Flavonoids, carotenoids, and α-tocopherol were discovered in significant amounts across a variety of seedling types. In particular, microgreens made from tartary buckwheat displayed a higher concentration of flavonoids than other microgreens. Both tartary and common buckwheat microgreens could serve as a healthy substitute for other vegetables [97]. Microgreens, sprouts, and leafy greens, as well as all phases of buckwheat, are excellent sources of phenolics, such as rutin, quercetin, vitexin, isovitexin, orientin, isoorientin, and chlorogenic acids, which give buckwheat its potent antioxidant properties. Research studies have also shown that microgreens can serve as a reliable source of bioactive compounds when compared to five different types of Brassica vegetables [98]. Xiao, Lester, Luo, and Wang [9] determined the concentration of ascorbic acid, carotenoids, phylloquinone, and tocopherols in 25 commercially available microgreens and identified a wide range of phylloquinone (Vitamin K_1_), from 0.6–4.1 μg/g fresh weight, and a 40 times higher concentration of Vitamin E in red cabbage microgreens (24.1 mg/100 g) compared to its mature counterpart (0.06 mg/100 g) of fresh weight. A study by Huang et al. [99] found notably higher concentration of glucosinolates in red cabbage microgreens than in their mature counterparts. In contrast, a previous study shows lower sugar concentrations in pepper cress and red amaranth microgreens than in mature plants [7]. In a study of 20 celery genotypes, macro- and micro-nutrients concentrations were compared, and it was found that concentrations of N, P, Na, Ca, and S were higher in microgreens, whereas K concentrations were higher in mature leaves [100]. Different microgreens exhibit varying levels of nutrients, vitamins, and minerals shown in (Figure 5). Recent research showed a promising level of essential fatty acids, oleic acid, linoleic acid, and terpenes in *Brassicaceae* microgreens [101]. In the early stages of plant growth, microgreens offer a wide range of sensory attributes, such as appearance, colours (crimson, green, multi-coloured, red, purple), flavour (neutral, pungent spicy, sweet, slightly sour), and texture (crunchy, tender, juicy) [29]. The most effective means of assessing human perception is to administer human sensory tests.

**Figure 6:**
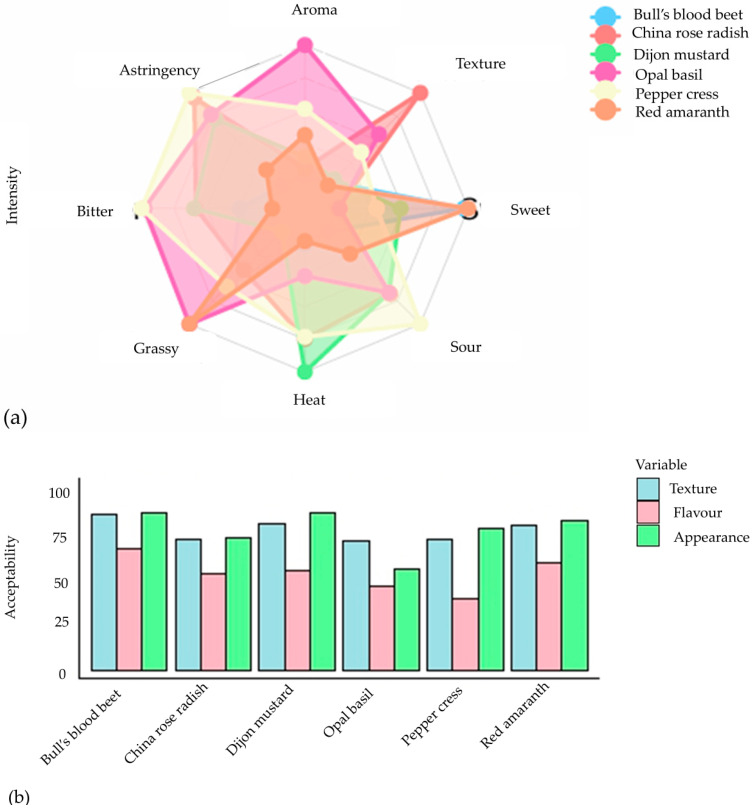
The evaluation conducted by the consumer panel focused on the intensity (**a**) and acceptability (**b**) of sensory qualities of six microgreens (rating 0 to 100) [102].

These tests were conducted through an organoleptic system that provides detailed information on the sensory characteristics of a food product. The assessment of sensory attributes among six different types of microgreens demonstrated a notable preference for visual appearance and texture, as indicated by scores surpassing 70, except for opal basil, which obtained a satisfactory rating (Figure 6a). Overall, the acceptability of flavor was deemed adequate, notwithstanding peppercress, which exhibited the least favorable eating experience due to pronounced bitterness and astringency (Figure 6b). Bull’s blood beet and red amaranth emerged as the top performers, garnering the highest composite ratings in terms of visual appeal, texture, and taste, while also displaying minimal bitterness. Dijon mustard, possessing a sensory profile akin to that of bull’s blood beet and red amaranth, similarly received commendable ratings. These results underscore the significance of flavor characteristics, particularly astringency and bitterness, in determining the appeal of microgreens, thereby supporting prior research that associates elevated glucosinolate levels with reduced consumer acceptance [102]. As a result, human sensory evaluation has become a crucial aspect of the modern food industry, particularly for determining consumer acceptability, quality control, and creating new products.

## 4. Agronomical Benefits of Microgreens

### 4.1. Short-Growing Time

Microgreens are fast-growing and are usually harvested 7–21 days (Table 2) after the emergence of the first pair of true leaves [4,103]. While microgreens are ready to harvest in their early stages, seedlings can offer the same or a greater amount of nutrients than their mature counterpart. Due to their short life cycle, microgreens can be grown with or without nutrient supplements. Different microgreens have different harvesting days to attain optimal hypocotyl length (2.5–7.5 cm), ensuring maximum economic benefit. Mature broccoli production requires 100–150 days at the commercial level; however, broccoli microgreens can be ready in 7–9 days, which reduces the production time by 93–95% when compared to mature vegetables. Additionally, the nutrient concentration of broccoli microgreens was found to be 1.73 times higher than its mature counterpart [41,104].

### 4.2. Carbon Footprint

Growing microgreens uses a scientifically grounded pathway to reduce carbon footprint by employing efficient land utilisation, reducing water usage, optimising energy efficiency, and promoting localised production and distribution due to their short growing cycle and shelf life. Traditional farming practices impact the environment in various ways, including global greenhouse gas emissions, diminished soil fertility, lower crop yields, the direct impact of chemical fertilisers on marine, freshwater, and land ecosystems, deforestation, disruption of biodiversity, and habitat fragmentation [108,109,110]. Additional activity related to traditional crop practices is transportation to the end consumer. Produce cultivation in the greenhouses is a common agricultural practice, which causes one-third of total greenhouse gas emissions [111]. In addition, microgreens cultivation can occur at home without any special need for agricultural land, fertilisers, or harmful pesticides [12].

### 4.3. Energy Conservation

Greenhouse-grown fresh produce has been popularised due to its higher yield and better quality than traditional crop production [41]. However, greenhouses contribute to approximately 74% of the total energy consumption associated with crop production [111]. Growing microgreens is relatively quick and requires only a small greenhouse space per unit of crop. It also utilises natural light sources or LEDs, which are a low energy light source. Higher water consumption can be associated with field-related crop production [112]. Therefore, considering water usage for a sustainable cropping method is critical. A study demonstrated 158–236 times reduced water use in microgreens production compared to the mature vegetable, with equivalent nutritional value in both crops [41].

### 4.4. Higher Productivity

Agricultural production can be augmented through two primary methods: extensification, which involves expanding land area for crops, and intensification, which includes enhancing yields through increased inputs, better agronomic methods, advanced crop varieties, and innovative technologies to satisfy the world’s food requirements [108]. Microgreens can provide higher yields in shorter time frames since they can be cultivated intensively and harvested within one to three weeks [113]. Their production facilities can vary from small greenhouses to large farms. A properly managed microgreens production unit e.g., 400 m^2^, can produce approximately 90 kg of microgreens per week [6]. Murphy and Pill [24] concluded that pre-sowing of seeds in vermiculite enhanced shoot fresh weight by 21% when compared to sowing untreated seeds in rocket microgreens. Seeding density has a direct impact on microgreens productivity; however, it is not well optimised at the commercial scale to date and requires further research. Many growers recommend a thick seeding (over seeding) density to maximise production [6].

### 4.5. Food Security

Throughout the COVID-19 pandemic, many regions around the world were faced with the challenge of food insecurity [114]. The major benefit of controlled environment agriculture is that it is an alternative to traditional agriculture cultivation. CEA may resolve emerging problems like feeding the growing population through new production practices [115], and although yields are low, nutrient value is high. Microgreens can be successfully grown at home to supply an adequate amount of nutrients in case of an emergency [29]. Commercially, all-year-round production and high-selling prices make them a great option for urban farming. Additionally, microgreens can act as excellent tools for nutrition awareness in academia and for farmers [12].

### 4.6. Minimising Waste

Microgreens can be produced using several different growing media (Table 1) or hydroponically [41]. Microgreens use nutrients present in the growing media to build plant biomass [111]; however, any nutrients remaining in compost are wasted when this is discarded at the end of crop production. In contrast, microgreens cultivated hydroponically flourish in nutrient-dense water solutions, which obviates the necessity for soil as a growing medium. Producing microgreens by this method reduces waste as a low quantity of water is utilised and fertilisers can be recycled in the solution [116].

## 5. Future of Microgreens

### 5.1. Comprehensive Nutritional Assessment

It is necessary to evaluate the nutritional content of microgreens holistically rather than by concentrating on certain chemical families. Untargeted metabolomic research could provide insight into the intricate makeup of nutrients and antinutrients [117]. The nutritional value and health advantages of microgreens, baby leaves, sprouts, and fully-grown plants differ significantly depending on the species, growth stage, and cultivation methods. While numerous studies have emphasised the nutrient-rich nature of microgreens, additional research is necessary to evaluate their nutritional profiles against those of mature plants from the same species cultivated and examined under similar conditions. Further investigation is also required to fully utilise the opportunity that CEA’s growth environment presents to produce targeted phytonutrients in microgreens.

### 5.2. Increased Demand and Market Growth

Microgreens are expected to become more and more well-liked because of their distinctive flavour profiles and highly nutritious content. There will probably be a rise in demand for these specialist greens as customers become more health-conscious [118]. The emerging trend of microgreens in the market is expected to expand further, with new products and applications such as drinks and meals enhanced with microgreens [119].

### 5.3. Advancements in Cultivation Techniques

The use of CEA methods, the concepts of hydroponics and aeroponics, will become more widespread. These methods enable continuous production throughout the year, decrease water and energy usage, and enhance crop yields. The integration of data analytics and machine learning will enhance monitoring and automation in microgreens cultivation, leading to more efficient and sustainable farming practices [120]. Artificial Intelligence (AI) can be employed to design an effective image acquisition process that considers both environmental factors and the growth stage of the microgreens. Additionally, a matrix chromogenic array combined with AI may be used to create quick detection platforms to identify the early development of plant and human infections [121].

### 5.4. Environmental Impact and Sustainability

The emphasis on sustainable food production, especially in areas vulnerable to climate change, is expected to boost the use of microgreens. The reduced water and soil usage associated with indoor farming is also expected to be a significant contributing factor to the environmental benefits of microgreens [41]. Furthermore, using alternative growing media such as coconut coir and compost mixtures can lessen dependence on peat [42], thereby improving the environmental sustainability of microgreens production.

### 5.5. Policy and Regulatory Frameworks

An extensive survey of microgreens producers in the United States highlights the inadequate comprehension of food safety measures within the microgreens business [122]. A study conducted in the US shows that the Produce Safety Rule (PSR) of the Food Safety and Modernisation Act (FSMA) covers the safety of produce, including microgreens. However, some growers are uncertain about their obligations under the rule [123], indicating the challenges of this emerging sector. Another major challenge for microgreens growers in implementing effective risk management is the dearth of tailored training and resources, as many operate in residential areas. Many growers rely on informal sources for education, highlighting the need for centralised and accessible training content [123]. In addition, supporting microgreens initiatives requires integrating green practices into microfinance, especially in Europe where funding prospects for sustainable agriculture are being shaped by regulatory developments such as the EU Green Deal [124]. The expansion of the microgreens industry can be facilitated by green microfinance, which can motivate farmers to use environmentally friendly techniques [124]. A comparative study of Brazil and Germany underscores the shifting regulatory environment for microgeneration and mini-generation, which closely mirrors the development of microgreens in fostering sustainable agricultural methods [125]. Despite the potential benefits of microgreens for improving nutrition and promoting sustainability, the regulatory framework surrounding them is intricate and differs substantially among nations. This situation calls for continuous adaptation and the incorporation of lessons learned from global experiences. This sector requires attention from governments and regulatory bodies to develop guidelines and standards for microgreens production, ensuring food safety and quality. Policies will support the growth of urban farming and controlled environment agriculture, fostering a more resilient and sustainable food system.

## 6. Conclusions

Microgreens are a promising option for producing high-value crops, and optimizing cultural practices such as seed density, growing media, irrigation water safety, light quality and quantity, temperature, and relative humidity is essential for growing these nutritious horticultural crops. Research shows that factors like light quality and intensity, humidity, seed density, and the substrate type greatly affect the growth and nutritional content of microgreens. Customised growing techniques can improve the yield and nutritional content of these plants, positioning them as an advantageous option for urban farming and sustainable agricultural practices. They can be grown successfully in domestic settings and large vertical farms, although they do not provide bulk calories. However, they offer comparable or greater amounts of phytonutrients than their mature counterparts, with a lower environmental impact due to the use of recycled nutrient solutions, lower chemical inputs, and reduced land use. Ongoing research is crucial for discovering new plant species that can be cultivated as edible microgreens and for devising methods that lower production costs while boosting yields. By optimizing elements like seed density, growth mediums, and lighting within CEA systems, both the yield and nutritional quality of microgreens can be enhanced. By applying these insights, microgreens production can be enhanced, contributing to food security and human nutrition.

## Figures and Tables

**Table 1:** Impact of different growing media on various microgreens species.

Substrate	Crop	Observation/Challenges	Ref.
Natural Synedrella residues (aerial part of Synedrella nodiflora)	Beet	Better quality and yield of beet microgreens.	[36]
Soil, vermicompost, cocopeat, farmyard manure (FYM) water	Mustard	Higher plant weight reported in vermicompost.	[37]
Mixture of cocopeat, perlite, vermiculite and vermicompost	Kale, broccoli, lettuce, and turnip	Observed variation in the performance of different microgreens varieties.	[38]
Coconut, jute fibre, vermiculite	Green basil, red basil, rocket	A significant effect on yield and dry matter percentage of microgreens.	[8]
Agave fiber, capillary mat, cellulose sponge, coconut fiber, peat moss	Coriander, kohlrabi, Pak choi	The highest fresh and dry yield was obtained from peatmoss, although its dry matter and phenolic content were low. Nitrate and macro-nutrient levels rise when natural fibre substrates are used.	[39]
Rockwool, cocopeat, Merang paper, Hydroton	Broccoli	Observed better growth of broccoli microgreens on cocopeat supplemented with coconut water.	[40]
Peat, Sure to Grow^®^ textile fibers, jute-kenaf fibers	Rapini	Recycled textiles showed comparable fresh biomass yield as peat.	[29]
Vermicompost, micro-mat hydroponic growing pads	Broccoli	Microgreens on compost showed greater elemental concentration than when grown hydroponically.	[41]
Cannabis mat, coco coir and peat	Mustard, pea, and radish	Peat can be successfully replaced by coconut coir and cannabis mat for mustard microgreens.	[42]
Sphagnum peat, coconut coir	Radish	In comparison to coconut coir, sphagnum peat requires less fertiliser to produce the same amount of microbial load within safe and legal limits.	[43]
Coconut coir, vermiculite, jute	Green basil, rocket, and red basil	Both species and substrates affect yield and nutrition. For green and red basil, respectively, coconut fibre and vermiculite are the most effective.	[44]
Posidonia natural residue and peat	Mizuna and rapini	Posidonia leaves or fibres were added to microgreens to raise their and B content without adversely impacting their production.	[45]
Beifiur^®^ S10, Carolina Soil^®^ organic, Carolina Soil^®^ seedling, and CSC^®^ vermiculite	Purple cabbage	No impact of substrate type on shoot height at harvest or shoot fresh/dry matter yield.	[46]
Hemp mat, jute mat, Micro-Mats (wood fiber), Biostrate^®^ (felt fibre)	Broccoli, cabbage, kale, mustard, and radish	Fresh, dry shoot weights and mineral nutrients in the microgreens under examination were affected by the kind of substrate. Microgreens in hemp had the highest weight, height, and K concentration, but the lowest N concentration.	[47]

**Table 2:** Range of harvesting time (days) in microgreens species.

Species	Harvesting Day	Ref.
Radish ruby	Day 5	[105]
Cabbage Chinese	Day 6	[105]
Broccoli	Day 7	[41]
Red amaranth and leafy vegetable amaranth	Day 8	[64]
Arugula	Day 13	[24]
Lettuce	Day 15	[106]
Watercress	Day 17	[105]
Upland cress	Day 20	[105]
Parsley	Day 21	[107]

## Data Availability

Not applicable.
